# Spatio-temporal profiling of coastal hazard / vulnerability assessment along the Gujarat coast, India, over a period of 35 years

**DOI:** 10.1038/s41598-026-44855-0

**Published:** 2026-04-15

**Authors:** Chandra Shekhar Dwivedi, Ananya Modak, Manali Santra, Arvind Chandra Pandey, Prem Chandra Pandey, Debashis Mitra, Navneet Kumar, Komali Kantamaneni

**Affiliations:** 1 Department of Geoinformatics, Central University of Jharkhand, Ranchi, Jharkhand 835205 India; 2 Department of Life Sciences, School of Natural Sciences, Shiv Nadar Institution of Eminence (deemed to be University), Greater Noida, Uttar Pradesh India; 3 Marine and Atmospheric Sciences Department, Indian Institute of Remote Sensing, (IIRS-ISRO), Dehradun, Uttarakhand India; 4 Division of Ecology and Natural, Resources Management, Center for Development Research (ZEF), University of Bonn, 53113 Bonn, Germany; 5 National Institute of Disaster Management (NIDM), Ministry of Home Affairs, Govt. of India, Vijayawada, India; 6 School of Engineering and Computing, University of Lancashire, Preston, PR1 2HE UK; 7 UN SPIDER- UK, Regional Support Office, Preston, PR1 2HE UK

**Keywords:** Shoreline change, Sea level rise, Coastal Vulnerability Index (CVI), Gujarat coast, Geospatial modelling, Climate sciences, Environmental sciences, Natural hazards

## Abstract

Coastal ecosystems are highly dynamic and fragile, facing increasing threats from climate-induced changes, sea-level rise, extreme wave action, vegetation loss, and diverse human activities. These factors create immense pressure on fragile ecosystems and species richness and accelerate shoreline degradation and erosion. This study presents a comprehensive assessment of coastal vulnerability along the Gujarat which is longest coastline in India, using a multi-parameter Coastal Vulnerability Index (CVI). The analysis incorporated nine existing key variables, including shoreline change rate, sea level change, geomorphology, tidal range, elevation, slope, significant wave height, land use/land cover (LULC), and population density. These datasets are derived from satellite imagery, tide gauge data, stationed data, and socio-economic datasets. Results indicate that marked spatial variability in vulnerability: while 60% of Gujarat’s coastline falls under low to very low risk categories, approximately 15% is classified as very high vulnerability, concentrated in central districts such as Bhavnagar, Anand, and Morbi. Conversely, the north-western (Kachchh, Devbhumi Dwarka) and southern (Porbandar, Gir Somnath) stretches demonstrate greater resilience, aided by extensive mudflats, mangroves, and stable geomorphic features. Comparative shoreline changes analysis (1990–2025) revealed a reversal of earlier erosive trends, with 61% of transects now exhibiting accretion. Elevation, slope, and sea level rise emerged as the most influential drivers of vulnerability, while socio-economic factors (LULC and population density) amplified exposure in hazard-prone zones. This integrated CVI approach provides the most spatially detailed vulnerability mapping of the Gujarat coast to date, offering critical insights for policy and decision makers which help in climate adaptation, sustainable coastal zone management, and disaster resilience planning in this area.

## Introduction

Coastal regions are among the most dynamic and fragile environments on the planet. These transitional zones, where terrestrial and marine systems intersect, host a range of complex geophysical processes that support diverse ecological systems. However, the accelerating pace of sea level rise, driven by global climate change, is placing many of these sensitive habitats under serious threat. For instance, global mean sea level rose by a record 97 mm in 2021^1^. Rising sea levels, combined with intensified wave action, contribute to processes such as shoreline erosion, flooding, storm-surge amplification, coastal reconfiguration, sediment deposition, and even the submergence of river valleys and low-lying areas^2^. These changes in coastal morpho dynamics often lead to environmental vulnerabilities that are difficult to predict and mitigate^3^. In their study, Scott et al.^4^ reported that a one-meter rise in sea level could submerge nearly 29% of beachfront resort infrastructure across the Caribbean, underscoring the critical need for vulnerability assessment. Reliable data on coastal sensitivity are therefore fundamental to protecting both ecosystems and communities.

In recent years, the evolution of geospatial technologies, particularly Geographic Information Systems (GIS), has significantly advanced the ability to analyse and interpret coastal risks. Innovations in photogrammetry, digital image processing, and topographic modeling have further refined coastline detection methods^5,6^. As noted by Li and Ma^7^, coastlines are among the most distinct features of the Earth’s surface and are recognized by the International Geographic Data Committee as essential environmental attributes. Scientists have long focused on measuring changes along coastlines using both conventional cartographic techniques and modern computational tools. GIS and remote sensing, in particular, have become indispensable for their ability to quickly and affordably analyse large-scale spatial data. Numerous studies have used satellite imagery analysis, including pixel classification, band differencing, and threshold techniques, to identify shoreline shifts^8–10^. Accurate shoreline delineation requires the clear distinction of land and water in imagery, which has led to the development of several water indices. Tools like the Normalized Difference Water Index (NDWI)^11^, Modified NDWI^12^, Water Ratio Index (WRI)^13^, and Automated Water Extraction Index (AWEI)^14^ have proven effective in enhancing water feature detection. The field has since evolved toward automatic and semi-automatic shoreline extraction using a variety of algorithms. Alesheikh et al.^15^ introduced the concept of band ratio analysis for shoreline detection. Earlier works by Braud and Feng^5^, and Frazier and Page (2000) also leveraged Landsat imagery for shoreline mapping. Classification techniques, both supervised and unsupervised, have also been successfully employed^16,17^. More recently, Chen et al.^18^ proposed a novel approach that uses greenness and moisture bands from the tasselled cap transformation (TCT) to refine shoreline extraction.

Shoreline erosion remains a global concern, occurring at rates ranging from 0.01 to 10 m annually^19,20^. Studies employing both qualitative and quantitative approaches have documented spatiotemporal shoreline changes worldwide^21–24^. To quantify the rate of change, analytical tools like the Digital Shoreline Analysis System (DSAS), an ArcGIS extension, utilize statistical methods such as End Point Rate (EPR), Linear Regression Rate (LRR), and Weighted Linear Regression (WLR)^25–27^. For example, Nassar et al.^28^ used DSAS to examine shoreline changes along the North Sinai coast, Egypt. Similarly, Yan et al.^29^ documented an 8% erosion rate along the Yancheng coast in China. In Brazil, Santos et al.^30^ applied DSAS to analyse short- and long-term coastal dynamics near Joao Pessoa. Various models, such as those based on historical erosion rates, sediment dynamics, and static inundation mapping have been utilized to forecast potential risks^31^**.** Among these, the Coastal Vulnerability Index (CVI) has emerged as a widely accepted framework for assessing shoreline risk at the international, regional and sub-regional scales^32–^^34^. The Coastal vulnerability index was initially developed by Gornitz et al.^35^ and further refined and applied at the regional level by Thieler and Hammar^36^ to analyse and calculate coastal vulnerability. The CVI methods assess coastal system’s susceptibility to environmental stressors and hazards by assigning weighted values to key physical and socio-environmental indicators. This approach helps generate a relative measure of coastal risk, that reflects both the inherent adaptability of the coastal environment and its likely response to sea level rise. Several studies in India have applied CVI to evaluate vulnerability across maritime states, including Sagar Island and Bulcherry Island of Indian Sudarbans region WB^37–41^, Odisha^42,43^, Andhra Pradesh^44^, Tamil Nadu^45–47^, Kerala^45–51^, Karnataka^52^, Mumbai coast, Maharashtra^53^ and Gujarat^54–56^. Jayanthi et al.^57^ investigated shoreline changes and the impacts of sea level riseon the south-eastern coast. Ratheesh et al.^58^ conducted a comprehensive shoreline change analysis of the entire Indian coast, revealing that 1144 km of the entire Indian coast, revealing that 1,144 km had undergone erosion and 1084 km accretion, while 5321 km remained unchanged during the study period from 2004–2006 to 2014–2016. Additionally, Kankara et al.^59^ provided a detailed status report covering shoreline alterations across all Indian coastal states over 26 years (1990–2016). A geographic information system (GIS) and a multi-criteria decision (MCDM) method can be effectively integrated to assess coastal risk and vulnerability^60–62^. Recent methods show that integrating machine-learning models with CVI frameworks improves the weighting of key indicators such as shoreline change, sea-level rise, coastal slope, wave climate, and geomorphology. Studies from Java and Moroccan coasts ^62–64^ confirm that ML-assisted geospatial approaches better capture non-linear coastal processes, thereby strengthening vulnerability assessment under climate and anthropogenic stress. Similarly, Mary et al.^65^ has employed machine learning models such as SVM, random forest and decision tree to evaluate coastal vulnerability in the Chennai coast.

India, with a coastline stretching over 11,098.81 km, is highly vulnerable to coastal hazards^10,66^. Coastal regions have high population densities, and because they rely on abundant coastal resources, large populations are typically at serious risk from beach erosion and rising sea levels^67^. Yet vulnerability assessments remain underutilised, despite being cost-effective compared to the high costs of early warning systems. As in many rapidly urbanizing nations, the Indian coastline is witnessing accelerated growth in urban infrastructure, ports, and settlements. This underscores the need for robust predictions of shoreline retreat, land loss, and cliff recession key inputs for coastal management strategies. Accurate projections can also help assess the ecological impacts of habitat transformation. Comprehensive knowledge, continuous observation, and early detection of shoreline changes are necessary to comprehend coastal dynamics and processes^68^. To support territorial planning and climate adaptation strategies, spatially integrated datasets must be employed. In this context, Integrated Coastal Zone Management (ICZM) offers a strategic framework that balances developmental goals with environmental conservation. It facilitates coordinated actions across sectors to mitigate natural hazards, regulate land use, and promote sustainable resource management. By implementing ICZM, coastal communities can address environmental challenges more holistically.

The coastal region of Gujarat is climatically controlled by the southwest monsoon, regional aridity gradients, and open exposure to the Arabian Sea, resulting in pronounced spatial variability in rainfall and coastal energy conditions. Annual precipitation ranges from less than 300 mm in Kachchh to over 2,500 mm in southern Gujarat, with monsoon winds, high waves, storm surges, and occasional cyclonic disturbances strongly influencing coastal processes^69^. High erosion rates along the Gujarat coast come from the interaction of intense semi-diurnal tides, extreme tidal ranges in the Gulf of Khambhat, and strong tidal currents that destabilize shorelines^70–72^. These natural drivers are further amplified by relative sea-level rise, sediment imbalance, and anthropogenic activities such as infrastructure especially port development, dredging, and land reclamation, making the region highly vulnerable to coastal erosion^60,61^. The primary objective of this study was to develop a detailed, location-specific geospatial database identifying areas along a segment of the Gujarat coast that are most susceptible to coastal hazards. To achieve this, rank-based coastal vulnerability index analysis was carried out incorporating several key physical and socio-environmental parameters. These included rates of shoreline change, tidal fluctuations, wave height, coastal elevation, slope gradient, sea level variation, geomorphological features, land-use and land-cover patterns, and population density. Using these inputs, the Coastal Vulnerability Index (CVI) was calculated to assess the relative sensitivity of different coastal segments to future sea level rise. In addition to CVI mapping, cluster analysis was employed to rank coastal regions based on shared characteristics, providing deeper insights into spatial variability and the interaction between land and water boundaries. This integrated approach enhances the understanding of coastal vulnerability and supports more effective planning and management of high-risk coastal areas in Gujarat.

## Study area

Gujarat, one of the coastal states on India’s western coast, has the country’s longest coastline, covering a 10 km buffer area of approximately 17,352 km^2^, with a shoreline length of 1735.20 km^73^.

**Fig. 1:**
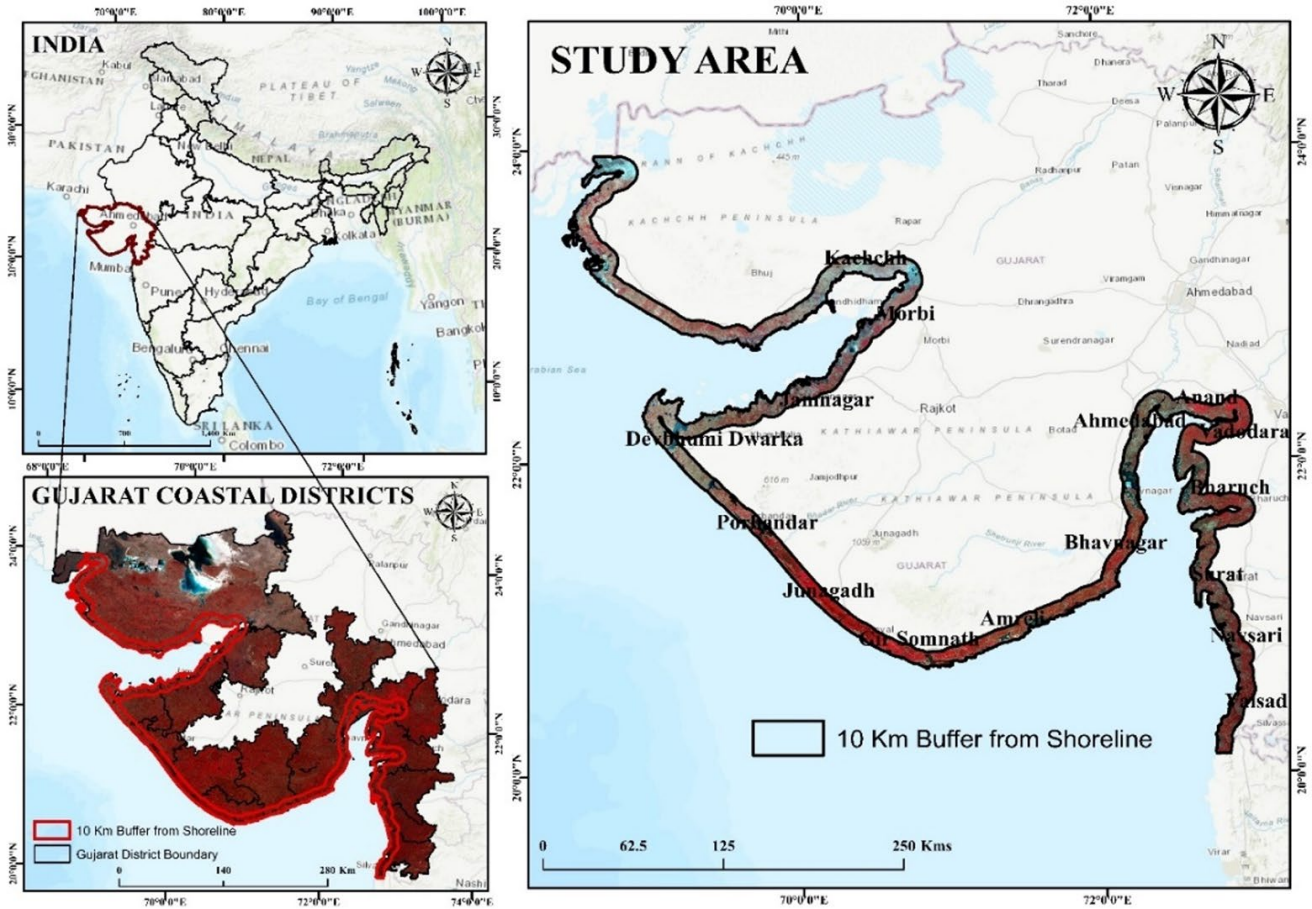
Location map of the study area. This figure was created by authors using ArcGIS 10.3.1 version on the Google Pro maps.

Geographically, the state lies between latitudes 20°10′N to 24°50′N and longitudes 68°40′E to 74°40′E. Much of Gujarat’s coastal terrain lies below the 30-m elevation contour, classifying it as low-lying and particularly susceptible to coastal erosion and flooding. The Gujarat coast is broadly divided into five distinct geomorphic regions: The Rann of Kachchh (1), the Gulf of Kachchh (2), the Saurashtra coast (3), the Gulf of Khambhat (4), and the South Gujarat coast (5). Two prominent gulfs, the Gulf of Khambhat and the Gulf of Kachchh, define much of the state’s coastal identity^73^. These gulfs are characterized by dynamic depositional and erosional patterns, driven by strong, semi-diurnal tides and high tidal ranges. The coastline supports diverse coastal ecosystems, including mangroves, mudflats, estuaries, islands, beaches, cliffs, and river deltas. The present study focuses on 16 coastal districts: Kachchh, Morbi, Jamnaegar, Devbhumi Dwarka, Porbandar, Junagadh, Gir Somnath, Amreli, Bhavnagar, Ahmedabad, Anand, Vadodara, Bharuch, Surat, Navsari, and Valsad, as shown in Fig. 1. Gujarat also plays a pivotal role in India’s coastal economy, contributing over 70% of the nation’s salt production^74^. The region’s climate varies significantly, with annual rainfall ranging from 300 mm in Kachchh to approximately 2,500 mm in southern districts.

### Data sources and methodology

This part covered the data, data sources, and techniques used to accomplish the research objectives. The procedures for collecting and preparing the data were outlined in detail, as were the techniques used to obtain crucial information and achieve the study’s goal. Data sources and Method are the two subheadings that provide information about the data and information used. In contrast, the Method subheading explains the strategies and procedures employed in the present study.

### Data sources

The study employed multi-temporal Landsat satellite imagery to examine coastline shifts during the last three decades (1990–2025). To track the movement of the coastline, this study looked at four distinct scenes at 10 and 15-year intervals (1990, 2000, 2015, and 2025). Based on the satellite’s path/row number over the Gujarat coast, Landsat TM and OLI satellite images were retrieved, and the relevant tidal data were supplied by the Permanent Service for Mean Sea Level (PSMSL). The 30-m-resolution images from Landsat 5 TM, Landsat 8, and Landsat 9 (1990–2025) were selected. Images with less than 10% cloud cover were used in order to minimize interference during analysis. The selected images underwent radiometric correction to account for atmospheric effects and sensor calibration, as well as geometric correction to align imagery with actual locations^75^. The exact dates of image acquisition were chosen to maintain stability in tidal phase, seasonal conditions, and cloud-free visibility over time. The selected images are all from the winter and early dry season (November-March), when spectral confusion between land and water is reduced by consistent vegetation cover and low tide heights. This seasonal consistency ensures reliable multi-temporal comparison and minimizes errors associated with phenological or hydrological variability. A detailed overview of the satellite imagery employed in this study, including acquisition dates, corresponding tidal conditions, and cloud cover percentages, is presented in Table 1.

**Table 1:** Dataset summary of satellite imagery acquisition parameters.

Satellite data	Path	Row	Date of acquisition	Cloud cover (%)	Time of the high tide	Tidal height (during high tide)	Resolution (m)
Landsat 5 TM	151	044	30/01/1990	0.95	6:24	6.0	
		045	30/01/1990	5.2	6:24	6.0	30
		043	30/01/1990	2.58	6:24	6.0	
	150	046	30/01/1990	0.29	6:24	6.0	
		044	07/02/1990	0.16	2:46	5.3	
	149	044	16//01/1990	0.83	5:32	5.7	
		045	16/01/1990	4.2	5:32	5.7	
		046	16/01/1990	1.3	5:32	5.7	
	148	045	28/02/1990	0.13	4:45	6.1	
		046	28/02/1990	1.22	4:45	6.1	
Landsat 5 TM	151	043	09/02/2000	0.59	4:48	5.9	
		044	04/12/1999	0.55	8:11	5.0	30
		045	04/12/1999	1.4	8:11	5.0	
	150	045	30/01/2000	0.4	9:06	4.6	
		044	11/11/1999	3.01	4:08	5.7	
	149	046	23/01/2000	0.03	4:06	6.4	
		045	23/01/2000	1.0	4:06	6.4	
		044	23/01/2000	4.5	4:06	6.4	
	148	045	16/01/2000	2.43	8:57	5.0	
		046	16/01/2000	1.4	8:57	5.0	
Landsat 8 OLI	151	045	30/01/2015	4.6	14:03	4.8	
		044	19/03/2015	5.02	2:49	5.9	30
		043	30/01/2015	0.00	14:03	4.8	
	150	046	20/12/2014	0.8	3:47	5.4	
		045	23/01/2015	0.00	4:15	6.4	
		044	20/12/2014	1.2	3:47	5.4	
	149	045	16/01/2015	0.73	8:43	4.7	
		044	21/03/2015	6.7	2:53	6.2	
	148	046	09/01/2015	3.53	4:51	5.8	
		045	08/01/2015	1.45	4:21	5.8	
Landsat 9 OLI	151	045	24/12/2024	0.1	5:34	4.9	30
		044	24/12/2024	2.01	5:34	4.9	
		043	24/12/2024	0.05	5:34	4.9	
	150	045	02/01/2025	2.95	4:05	6.0	
		044	25/12/2024	3.48	9:34	4.8	
	149	046	04/01/2025	0.00	5:18	6.0	
		045	03/01/2025	5.01	4:41	6.0	
		044	19/12/2024	0.09	5:05	5.9	
	148	046	03/01/2025	6.02	4:41	6.0	
		045	03/01/2025	1.93	4:41	6.0	

### Methodology

**Fig. 2:**
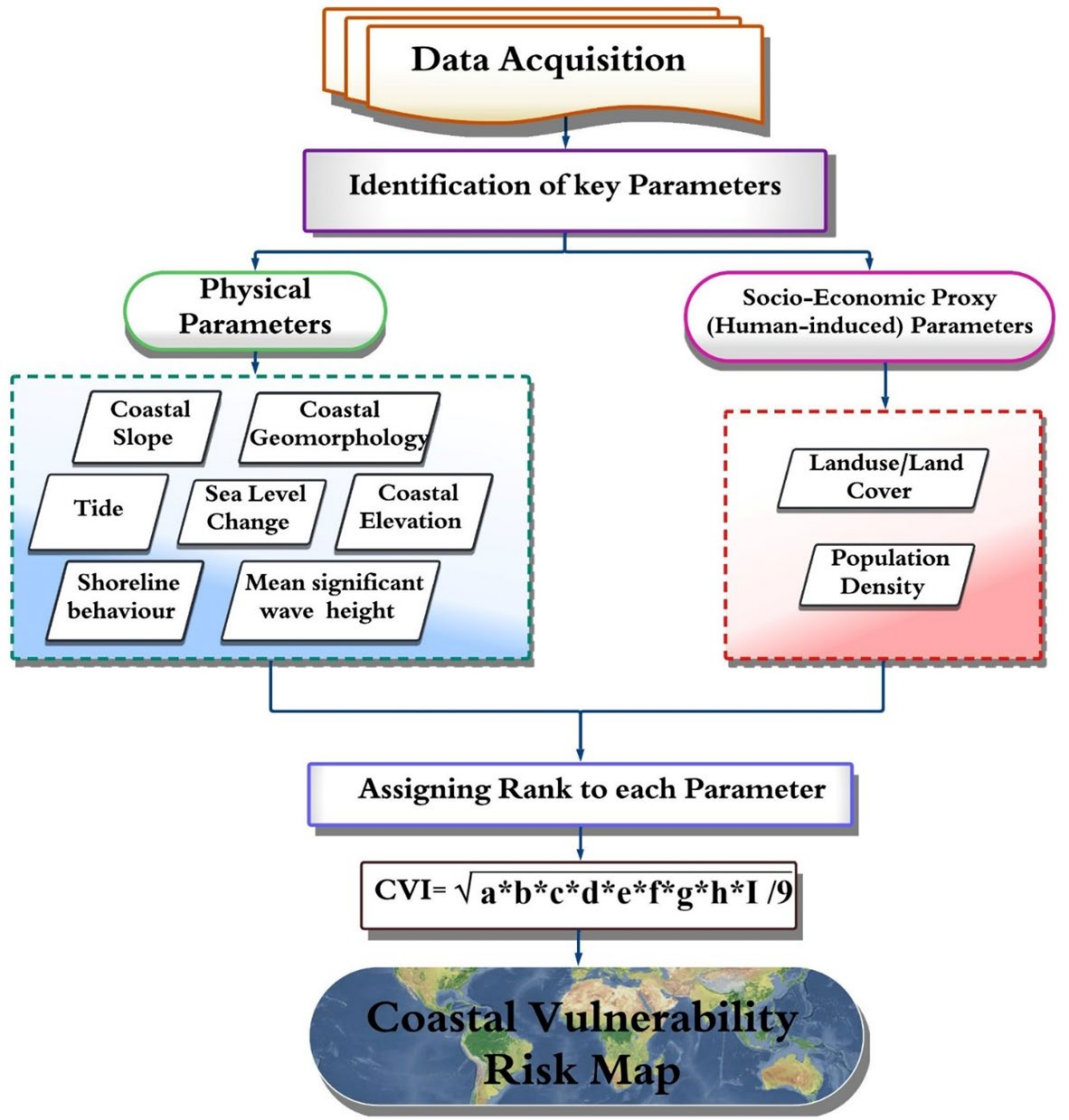
Schematic representation of coastal vulnerability assessment of the Gujarat coast.

Coastal vulnerability of the Gujarat coast has been assessed using seven physical parameters such as coastal slope, coastal geomorphology, tidal range, mean sea level, shoreline behaviour, coastal elevation, and significant wave height and two socio-economic proxy parameters (land use, land cover, population density—Table 2). Further, these variables were ranked on a common scale using a weighted method based on their contribution towards increasing coastal vulnerability^36,76,77^. A common scale is really helpful because it allows working with various vulnerability parameters on a comparable platform in a more flexible way. Rank has been assigned to each factor based on the information procured from earlier published literature^60,61,78^ and quantitative measurements of the variables, each variable is assigned a rating between 1 and 5. The Coastal Vulnerability Index (CVI) was then derived using Eq. (1), which calculates the square root of the product of the ranked variables divided by the total number of variables^36,77^. The flowchart of the CVI estimation method is shown in Fig. 2, and the various parameters used in CVI estimation are shown in Tables 3 and 4.

**Table 2:** List of additional data for generating various parameters.

Parameter	Data source	Sensor/Platform	Spatial resolution	Remarks	Number of studies referencing similar data
Shoreline change rate	Landsat 5, 8 & 9	USGS/NASA	30 m	Shoreline extraction and multi- temporal analysis of shoreline movement	^44,55,56,60,61,78^
Geomorphology	Bhukosh (Bhuvan-NRSC)	Topographic Maps	Vector-based	Landform classification and terrain mapping	^55,60,61^
Coastal slope	ASTER GDEM	Terra Satellite (NASA)	~ 30 m	Slope derived from elevation for vulnerability assessment	^55,60,61^
Regional elevation	SRTM	Shuttle Radar Topography Mission	~ 30 m (1 arc-sec)	Elevation data for flood and inundation analysis	^55,78^
Tidal range	WX-Tide Software	Modeled/Station Data	Station-based	Predicted tide levels for reference coastal locations	^55,60,61^
Significant wave height	Wave Watch III	NOAA/NCEP Model	Gridded (Global Model)	Open-ocean wave model output used for exposure estimation	^55,60,61^
Sea Level Change	PSMSL	Tide Gauge Observations	Station-based	Long-term sea level trends from global tide gauges	^55,78^
Land Use/Land Cover	Landsat 8	USGS/NASA	30 m	Classification of coastal LULC for spatial vulnerability assessment	^79,﻿78^
Population Density	GHSL	ASAR	3 arc sec	Estimates the resident population patterns of built-up areas	^55^

**Table 3:** Parameters and designated symbols/letters.

No	Designated symbol/letter	Parameter
1	**a**	Geomorphology,
2	**b**	Shoreline change rate (m/year),
3	**c**	Sea level change rate (mm/year),
4	**d**	Tidal Range (m),
5	**e**	Elevation (m),
6	**f**	Slope (degree),
7	**g**	Significant Wave Height (m),
8	**h**	Coastal LULC,
9	**i**	Population density

$${\mathbf{CVI}} = \sqrt {\mathbf {a*b*c*d*e*f*g*h*i}}/9$$

#### Creating spatial layers of the risk variables

##### Coastal geomorphology

The landforms that are more vulnerable to erosion and those that can aid in preventing it are identified by geomorphology^55^. Key landforms along Gujarat coastal region include alluvial plains, tidal flats, mudflats, salt pans, and Pediment-Pediplain complexes, along with mangrove swamps, creeks, aeolian plains, lagoons, beaches, and dissected hills^71^. Dissected hills, Wave cut platform and abandoned hill & cliff offer maximum resistance to erosion, and are much less susceptible, while soft sandy and muddy structures such as dunes, mudflats, mangroves, etc., offer the least resistance and are extremely vulnerable to sea level rise^80,81^. These features were identified and mapped using visual interpretation of satellite imagery. These rankings formed a foundational component in developing the Coastal Vulnerability Index (CVI), helping to quantify the relative risk along different coastal segments.

##### Shoreline extraction and shoreline change rate analysis

**Fig. 3:**
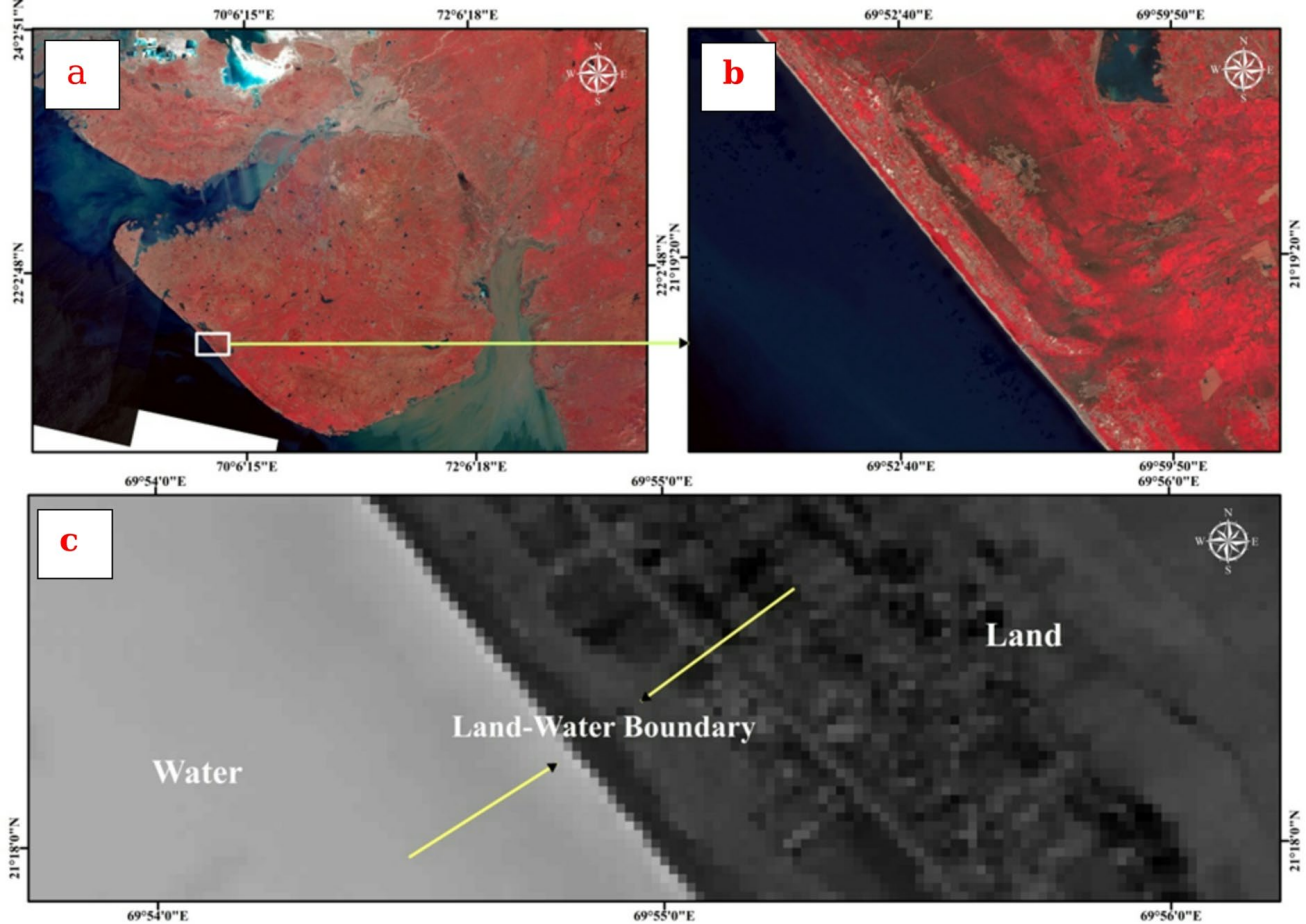
(a, b) Gujarat coast and its small subset is shown in RGB band and (c) shows the MNDWI for subset of Gujarat Coast, where black colour represents land areas and the grey colour shows water areas. These figures were created by authors using ArcGIS 10.3.1 version on the Google Pro maps.

**Fig. 4:**
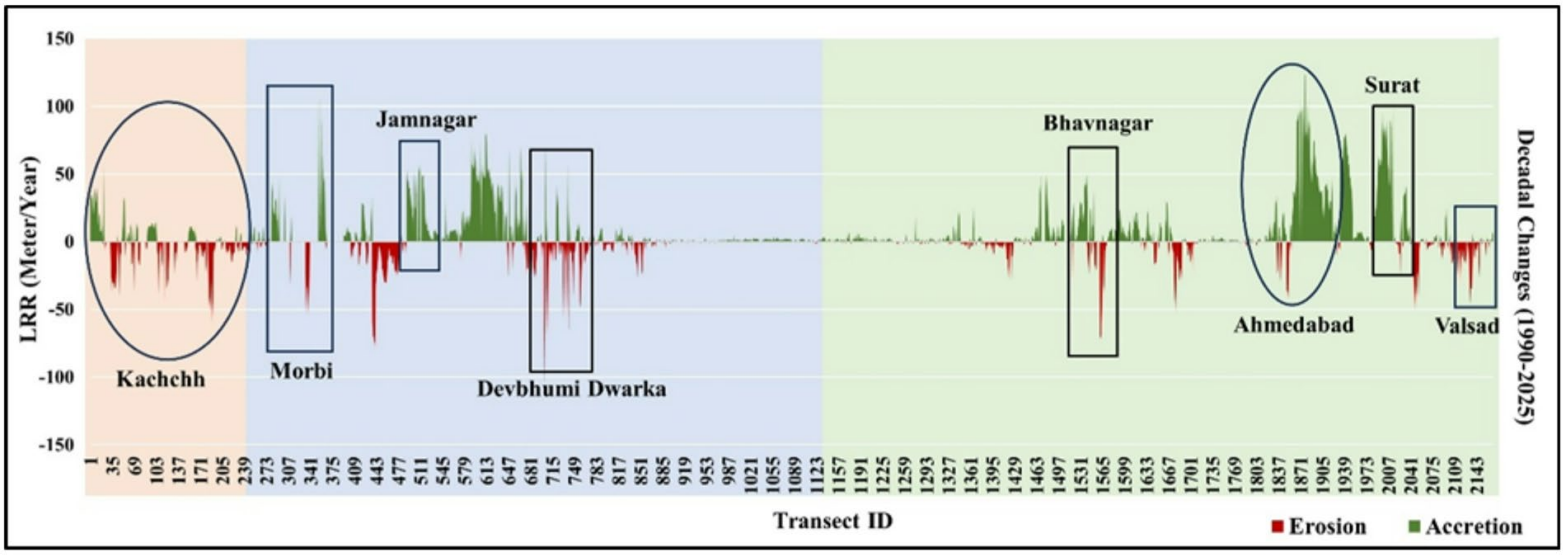
Decadal (35 years) shoreline changes based on Linear Regression Rate (LRR).

**Fig. 5:**
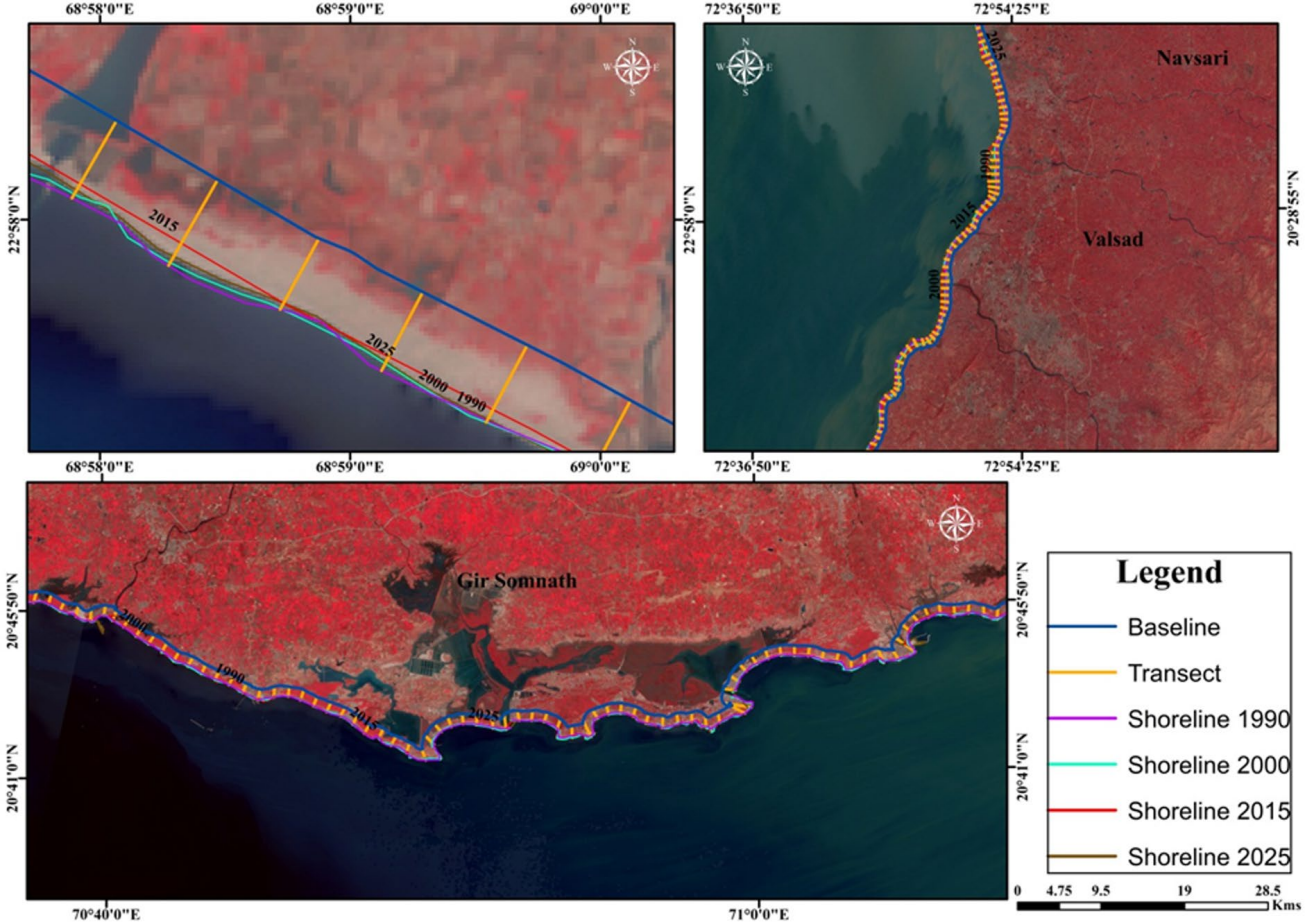
Transects, baseline and shoreline from 1990 to 2025. These figures were created by authors using ArcGIS 10.3.1 version on the Google Pro maps.

To assess the Gujarat coastline’s response to sea-level rise over the period of 35 years (1990–2025), shoreline positions from Multi-temporal Landsat TM, OLI imagery (30 m; USGS Earth Explorer) formed the primary dataset for shoreline delineation. An automated algorithm was developed on the Google Earth Engine (GEE) platform, wherein the Modified Normalized Difference Water Index (MNDWI) was first computed to enhance land–water contrast (Fig. 3). Dimensionality reduction via Principal Component Analysis (PCA) was applied to isolate water-related spectral variance. Subsequently, Support Vector Machine (SVM) classification generated binary land–water masks, on which zero-crossing and canny edge detection were implemented to extract shorelines. Accuracy was validated using the Sentinel-2 MSI image (2025) as high-resolution reference data. The extracted shorelines (1990–2025) were incorporated in ArcGIS (DSAS v5.0) to calculate statistical change metrics, namely End Point Rate (EPR), Net Shoreline Movement (NSM), and Linear Regression Rate (LRR) (Fig. 4). These Digital Shoreline Analysis System (DSAS) transect-based shoreline analyses^26,27^ enabled quantification of erosion–accretion patterns along the Gujarat coastal region (Fig. 5). These delineated shorelines for the four time periods (1990, 2000, 2015, and 2025) were imported into the DSAS tool within ArcGIS 10.8 for analysis. A baseline was also created parallel to the shorelines and added to the DSAS tool to support the calculations. To quantify shoreline change rate, a total 2,169 transects were drawn perpendicular to the baseline at 800 m intervals, using a smoothing distance of 2,500 m. Further, the Linear Regression Rate (LRR) technique was employed to measure long-term shoreline change over the 35-year period.

Shoreline change analysis was conducted using the Digital Shoreline Analysis System (DSAS), an extension of ArcGIS developed by the United States Geological Survey (USGS). The Linear Regression Rate (LRR) method was applied to calculate long-term shoreline change rates. This method estimates the rate of shoreline movement by fitting a least-squares regression line to shoreline positions through time. The reliability of the regression model was evaluated using the coefficient of determination (R^2^), which indicates the goodness of fit between shoreline positions and time. *The LRR method calculates the rate of shoreline movement by fitting a least-squares regression line to all available shoreline positions along each transect. The reliability of the regression model was evaluated using the coefficient of determination (LR*^*2*^*), which indicates the strength of the relationship between shoreline position and time. Shoreline change analysis was carried out along 2169 transects distributed along the Gujarat coast. The LR*^*2*^* value was calculated separately for each transect and therefore varies spatially depending on the temporal consistency of shoreline positions and local coastal conditions. In the present study, LR*^*2*^* values range from 0 to 1, with a mean value of 0.42 across all transects. Higher LR*^*2*^* values indicate a stronger linear relationship and greater confidence in the estimated shoreline change rate, whereas lower values suggest greater variability in shoreline behaviour. The lower LR*^*2*^* values observed in some transects reflect the dynamic nature of the coastline, which may be influenced by seasonal sediment transport, episodic storm events, and human activities along the coast. Areas exhibiting shoreline advancement due to sediment deposition were classified as low-risk zones, while zones experiencing retreat were marked as highly vulnerable*. Based on the calculated rates of change, shoreline segments were categorized into five risk classes: very low (1), low (2), moderate (3), high (4), and very high (5). This classification system provided a clear representation of erosion trends. It allowed the identification of the most hazard-prone sections of the coast^26,27^, serving as a critical input for regional coastal vulnerability assessments.

##### Sea level changes

The melting of glaciers and the thermal expansion of sea water are the main causes of sea level rise^55,69^. In this study, long-term sea level trends were examined using tide gauge data from the GLOSS, along with monthly mean tide records from Indian coastal stations. These historical datasets, covering nearly 100 years, provided valuable insights into regional sea level rise patterns. To minimize short-term variability from tides and wave action, average values were used. The collected data was used to create the graph with the year on the x-axis and sea level (mm) on the y-axis, thereafter the annual sea level change rate was computed using the slope of the graph (Table 4).

**Table 4:** Mean sea level rise (mm/Year) at selected stations.

Station	Latitude	Longitude	Sea level rise (mm/yr)
Okha	22.466	69.083	2.09
Kandla	23.016	70.216	2.13
Mangalore	12.916	74.800	2.27
Tupase	44.100	39.066	1.18

##### Tidal range

Tidal range is a key parameter in evaluating the vulnerability of coastal areas to both routine tidal inundation and extreme flooding events. Coastal regions experiencing significant tidal variation are considered highly vulnerable, as wide tidal ranges are associated with intense tidal currents that directly affect shoreline morphology and flooding potential^42,77,82–85^. The main factors causing the tidal range are storm surges and flooding^86^. The vertical difference between high and low tide is called tidal range. Data covering from 1990–2024 were obtained from WX Tide (https://wxtide32.informer.com/4.7/) for nine stations (Okha Point, Navabandar, Port Albert Victor, Bhavngar, Navinar Point, Khori Cheek, Hansthal Point, Navlakhi, Naviwat) are taken into consideration for analysing tidal range. The data is interpolated for the entire Gujarat coastline using the interpolation method. Further, interpolated data is used to classify the entire coastal region to five risk groups.

##### Coastal regional elevation

Understanding elevation is essential when evaluating the potential impacts of sea level rise on coastal regions, as it indicates how high the land lies above mean sea level. In this study, elevation data from the SRTM (https://search.earthdata.nasa.gov/search) was utilized to generate a detailed coastal elevation model. Geospatial methods are applied to the data processing. The geospatial tool is used to mosaic and clip the data along the coast. After converting the raster image to points, the elevation value is linked to the shoreline shape file. Risk has been assigned in to five classes considering that high-elevation areas of the coastline are less susceptible to sea level rise than low-elevated areas.

##### Coastal slope

The steepness of the coastline is described by its slope, which is measured in degrees in this work. The gentleness of the slope increases the amount of beach erosion and flooding^60,61^. In this study, slope values were derived from ASTER DEM data and analyzed using the Slope tool in Arc Map 10.8. The source of the ASTER DEM data is (https://search.earthdata.nasa.gov/search). Areas with gentler slopes were identified as high-risk zones, aligning with the methodology proposed by Rao et al.^82^. For consistency and comparison, all slope values were standardized and reported in degrees.

##### Mean significant wave height

The significant wave height helps identify areas more vulnerable to erosion because the wave energy responsible for the transportation of sediment increases with the square of the significant wave height^60^. Significant wave height data is taken from the National Weather Service Organisation (NWSO), Data processing was performed in R Studio software to compute mean values, subsequently converted from raster to point data, and finally processed using interpolation tools. Higher wave heights typically indicate stronger energy levels, which can accelerate coastal erosion and increase the risk of land submergence.

##### Land use /land cover

Land use/land cover (LULC) classification was carried out using Landsat-9 OLI imagery (30 m spatial resolution) for the year 2025. A supervised classification approach employing the Maximum Likelihood Algorithm was applied, and seven LULC classes, water bodies, mudflats, vegetation, agricultural land, built-up areas, barren land, and salt pans were identified. For each class, 115 training samples were selected across the study area to capture spatial variability. Classification accuracy was assessed using an independent validation dataset and a confusion matrix, resulting in an overall accuracy of 92% and a Kappa coefficient of 0.89, indicating strong agreement between the classified output and reference data and confirming the reliability of the LULC dataset for coastal vulnerability assessment.

##### Population density

The most important study parameter and the largest socioeconomic factor considered is population density. High population concentrations in coastal areas often reflect increased pressure on fragile ecosystems, leading to accelerated erosion and environmental degradation^87, 88^. In this study, village-level population density data were sourced from the GHSL database to evaluate the extent of human presence along the Gujarat coastline.

#### Risk rating

All nine criteria were categorised using the five risk levels: very low, low, moderate, high, and very high. The ranges utilised to group the risk rates for each parameter are shown in the following Table 5.

**Table 5:** Categorization and risk rating of input parameter.

Parameter	Coastal vulnerability rank				
	Very Low (1)	Low (2)	Moderate (3)	High (4)	Very High (5)
Coastal Geomorphology	Highland, Rocky & cliffy coast	Coastal plain, alluvial plain & pediment-pediplain complex	Supra and High tidal flat, Salt Pan, Channel Bar, Dam & Reservoir	Waterlogged, Low depression, Lagoon, Creek Network	Beach, Mangrove swamp, Mud flat
Shoreline change rate (m/y)	< 5	1 to 5	-1 to 1	-5 to-1	> -5
Sea level change rate (mm/y)	< 2.09	2.09 to 2.11	2.11 to 2.12	2.12 to 2.13	> 2.13
Tide range (m)	< 3.87	3.88 to 5.26	5.27 to 6.67	6.68 to 8.11	> 8.12
Elevation (m)	> 10	10 to 7	7 to 4	4 to 2	< 2
Coastal slope (degree)	> 32	32 to 24	24 to 16	16 to 8	< 8
Mean significant wave height (m)	< 0.96	0.97 to 1.06	1.07 to 1.17	1.18 to 1.28	> 1.29
Landuse Landcover (km^2^)	Forest, Mangrove Swamp	Barren Land, Salt Pan	Agricultural Land	Waterbody, Rann	Settlement
Population density (P/ km^2^)	< 3	3–25	25–45	45–60	> 60

#### Computation of coastal vulnerability index (CVI)

The Coastal Vulnerability Index (CVI) provides a quantitative framework to assess the relative sensitivity of shorelines to physical changes driven by future sea level rise. In this study, CVI values were calculated across the entire research area using a gridded approach, with each grid cell measuring 1*1 km. The shoreline was classified into five categories of vulnerability: very low, low, moderate, high, and very high. Each input variable, such as elevation, slope, tidal range, wave height, geomorphology, LULC, and population density, was ranked on a linear scale from 1 to 5, reflecting increasing susceptibility to coastal hazards. The composite CVI was then derived by calculating the square root of the product of these ranked variables divided by the total number of parameters. This method allowed for a consistent comparison of vulnerability across coastal segments. To assign CVI values spatially, vector-based algebraic operations were performed in ESRI ArcMap, enabling the visualisation and analysis of risk patterns along the Gujarat coastline. All CVI parameters are shown in Table 6.

**Table 6:** Shoreline length distribution across each vulnerability class for the nine selected variables.

Variable	Shoreline length in km (% share in bold) in each of the vulnerability classes (rank) Vulnerability Rank				
	Very Low (1)	Low (2)	Moderate (3)	High (4)	Very High (5)
Coastal Geomorphology	134 (6.20)	606 (37.31)	338 (20.28)	119 (5.51)	538 (30.69)
Shoreline (m/y)	400 (23)	298.4 (17)	563 (32)	197.6 (11)	276 (16)
Sea Level change rate (mm/y)	334 (22.34)	57 (3.55)	702 (37.01)	185 (10.94)	457 (26.17)
Tide Range (m)	350 (20.76)	594 (32.01)	436 (24.72)	218 (13.42)	137 (9.09)
Coastal Elevation (m)	269 (14.81)	196 (11.07)	290 (15.88)	639 (38.73)	341 (19.52)
Coastal Slope (degree)	154 (8.41)	191 (10.31)	432 (24.73)	619 (36.89)	339 (19.65)
Mean Significant Wave Height (m)	164 (10.34)	172 (10.71)	288 (15.60)	235 (13.15)	876 (50.21)
Land use/ Land cover (km^2^)	543 (29.75)	165 (10.69)	476 (26.65)	120 (8.33)	431 (24.57)
Population Density (P/km^2^)	109 (7.62)	784 (41.33)	361 (21.55)	293 (18.37)	188 (11.13)

## Results

First, the risk rate comparison and assessment of the coastal vulnerability parameters were conducted. An evaluation of the obtained CVI results was conducted in the subsequent section.

### Coastal geomorphology

**Fig. 6:**
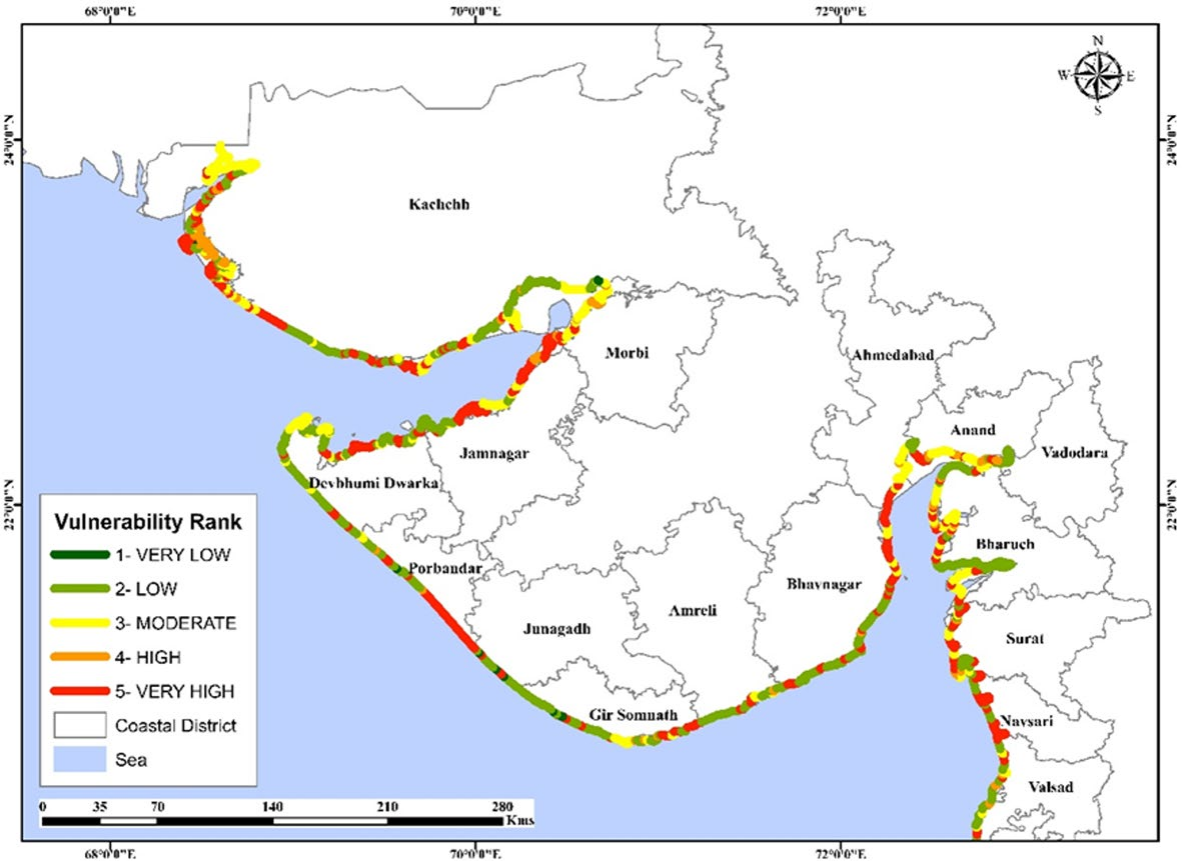
Coastal vulnerability map of coastal geomorphology for the Gujarat coast. This figure was created by authors using ArcGIS 10.3.1 version on the Google Pro maps.

The study area is largely dominated by the highly fragile Pediment-Pediplain (PP) Complex, which plays a significant role in defining the geomorphological vulnerability of the Gujarat coast. The spatial extent of these zones is illustrated in Fig. 6. Based on the vulnerability assessment of coastal geomorphology, approximately 37.31% of the coastline falls under the low vulnerability category, suggesting relatively stable landforms. In contrast, 30.69% of the transects exhibit very high vulnerability, indicating zones composed of weak, easily erodible geomorphic features. Moderate vulnerability was observed in 20.28% of the coastline, while 5.51% and 6.20% fall under high and very low vulnerability categories, respectively. The coastline changes a lot in its trend, shoreline features, and nearshore and offshore conditions, which shows that there is a lot of structural control^69^**.** Though the coast of Saurashtra is mainly made up of dunes, cliffs, Mud-tidal flats (Fig. 18a), estuaries, sandy beaches, and bays with elevated beaches close to Porbandar and Veraval^89^.

### Shoreline change rate

**Fig. 7:**
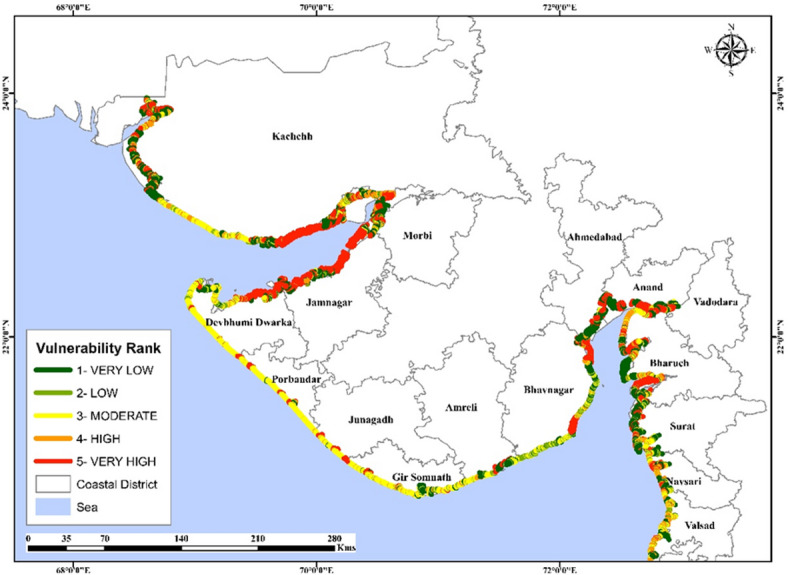
Coastal vulnerability map of shoreline change rate for the Gujarat coast. This figure was created by authors using ArcGIS 10.3.1 version on the Google Pro maps.

The historical rate of shoreline change shows the overall trend but cannot be used as a constant to forecast how the coast will behave in the future^82^. Conducted^60, 61^ a study using remote-sensing satellite data from 2001 to 2011. Coastal erosion was found to be a serious issue in South Gujarat’s coastal regions. The general patterns of shoreline behaviour offer valuable insights into how the coast may respond to future sea level rise. As shown in Fig. 7, shoreline changes over the past 35 years were analysed to assess erosion trends in the study area.

According to the findings, 8.80% of transects fall within the high vulnerability category, while 16.97% are classified as very high vulnerability zones, indicating active and severe erosion. Conversely, 16.77% of transects exhibit very low vulnerability, and 7.08% show low vulnerability. The largest share, 26.65%, falls into the moderate category, reflecting relatively stable coastal conditions. Overall, results indicate that accretion has slightly outpaced erosion from 1990 to 2025.

### Sea level change

**Fig. 8:**
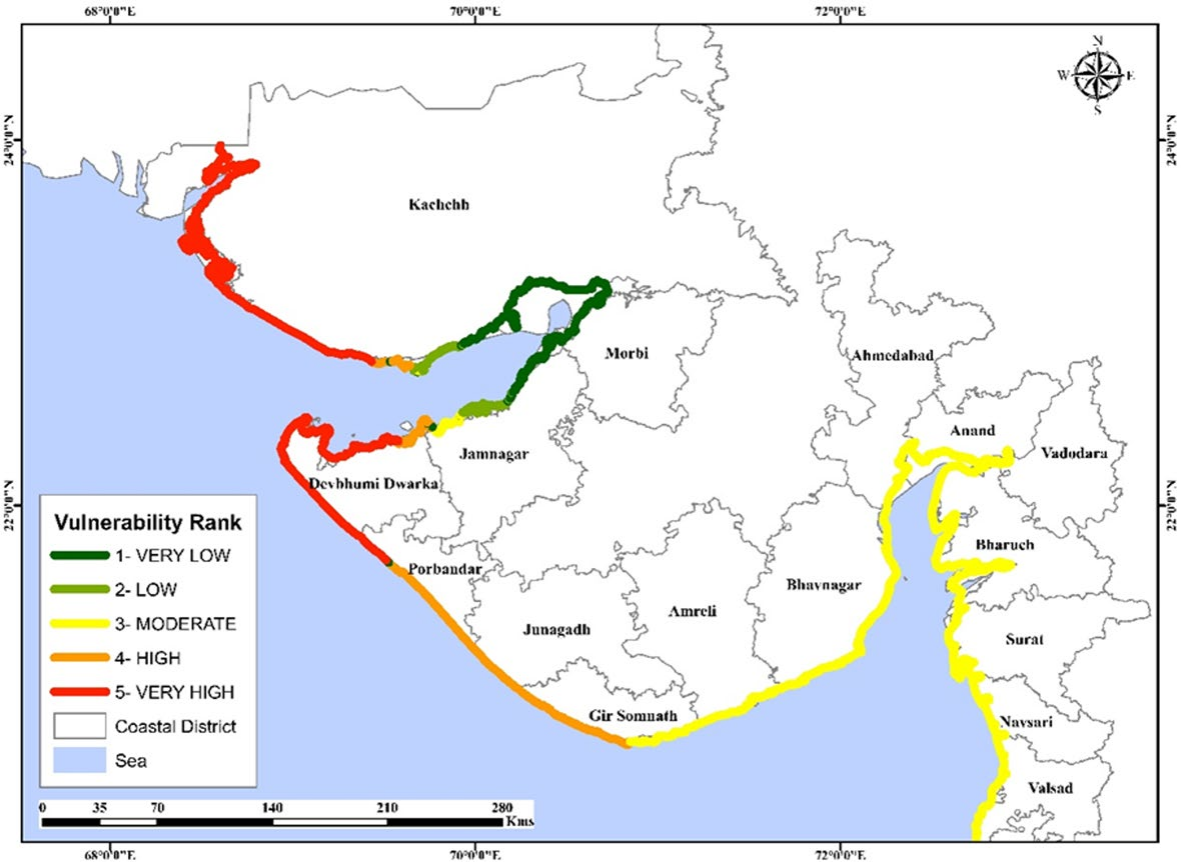
Coastal vulnerability of sea level rise for the Gujarat coast. This figure was created by authors using ArcGIS 10.3.1 version on the Google Pro maps.

The relative distribution of the vulnerability class for sea level fluctuations throughout the Gujarat coastline, Fig. 8. With 37.01% of transects falling into the moderate category, the sea level rise vulnerability assessment indicates widespread medium sensitivity. A noteworthy 26.17% exhibit very high vulnerability, signifying regions that are more vulnerable to the effects of sea level rise. In contrast, 10.94% and 3.55%, respectively, fall into the high and low groups, while 22.34% are in the very low category. These findings highlight the necessity of targeted adaptation measures in high-risk areas by indicating that a sizable section of the Gujarat coast may experience moderate to severe effects from sea level rise.

### Tidal range

**Fig. 9:**
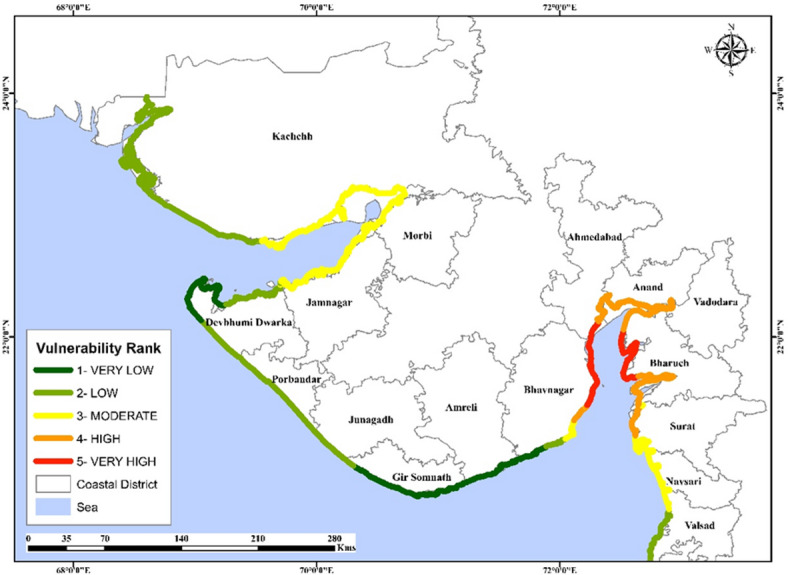
Coastal vulnerability map based on tidal range for the Gujarat coast. This figure was created by authors using ArcGIS 10.3.1 version on the Google Pro maps.

The Gujarat coast is characterised by semi-diurnal tides exhibiting pronounced diurnal inequality, with tidal amplitudes varying spatially along the coastline. Observed tidal heights typically range from approximately 4.8 m during lower tidal conditions to about 6.4 m at peak tidal levels, reflecting the strong tidal forcing that significantly influences shoreline behaviour in the region. Based on the mean tidal range analysis, 9.09% of transects are categorised as very high vulnerability and 13.42% as high, reflecting areas significantly influenced by tidal fluctuations. In contrast, low and very low vulnerability zones comprise 32.01% and 20.76%, respectively, while 24.72% fall into the moderate category, suggesting generally stable tidal conditions across much of the coastline (Fig. 9).

### Coastal elevation

**Fig. 10:**
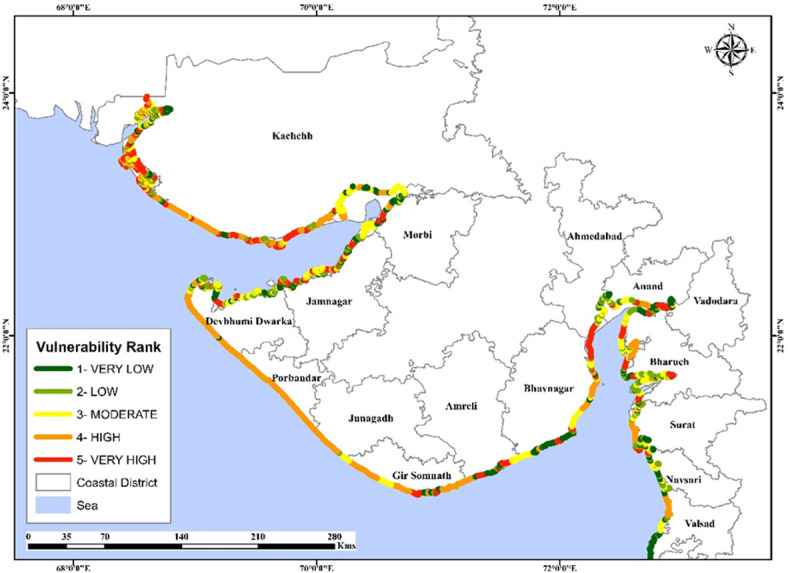
Coastal vulnerability map based on coastal Elevation for the Gujarat coast. This figure was created by authors using ArcGIS 10.3.1 version on the Google Pro maps.

Based on the slope analysis, 41.23% of transects along the Gujarat coast fall into the low vulnerability category, while 21.70% are classified as moderately vulnerable. High and very high vulnerability zones account for 12.65% and 15.14%, respectively, indicating regions with steeper coastal gradients (Fig. 10). The limited extent of flat terrain is reflected by only 9.28% of transects being categorised as very low vulnerability, suggesting that slope significantly influences the shoreline’s sensitivity to natural processes.

### Coastal slope

**Fig. 11:**
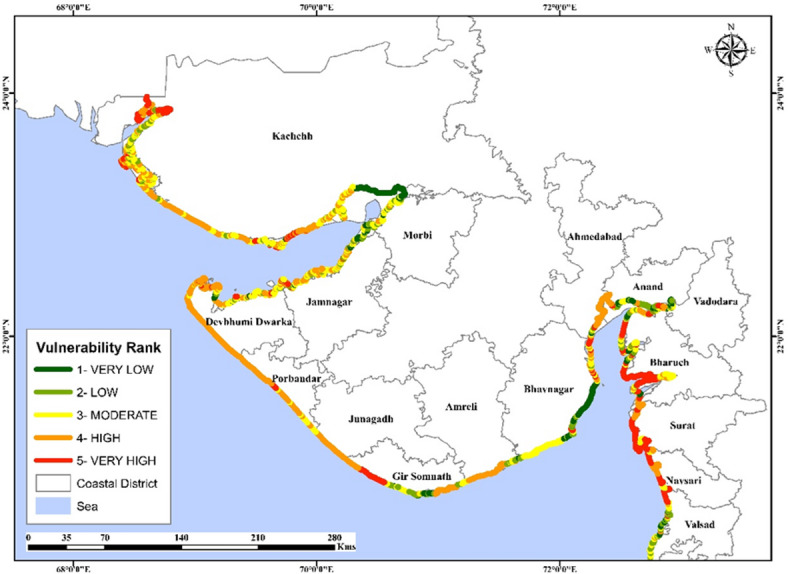
Coastal vulnerability map based on coastal slope for the Gujarat coast. This figure was created by authors using ArcGIS 10.3.1 version on the Google Pro maps.

Shoreline erosion and flooding are more common on gentler slopes^60,61^. The amount of land lost to flooding depends only on the slope: the lower the slope, the more land is lost. Rao et al.^82^ say that the coastal slope is the most important factor to consider when assessing how sea-level rise will affect a given coast. The slope-based coastal vulnerability assessment reveals that 19.08% of the transects fall into the high vulnerability category, while an even greater share 26.05% is considered very high vulnerability. Meanwhile, 28.73% of transects demonstrate moderate vulnerability. In contrast, only 15.80% and 10.35% of the transects fall into the very low and low vulnerability zones, respectively. These findings suggest that stable coastal zones are limited, reinforcing the importance of slope analysis in understanding flood risk and informing shoreline management strategies (Fig. 11).

### Significant wave height

**Fig. 12:**
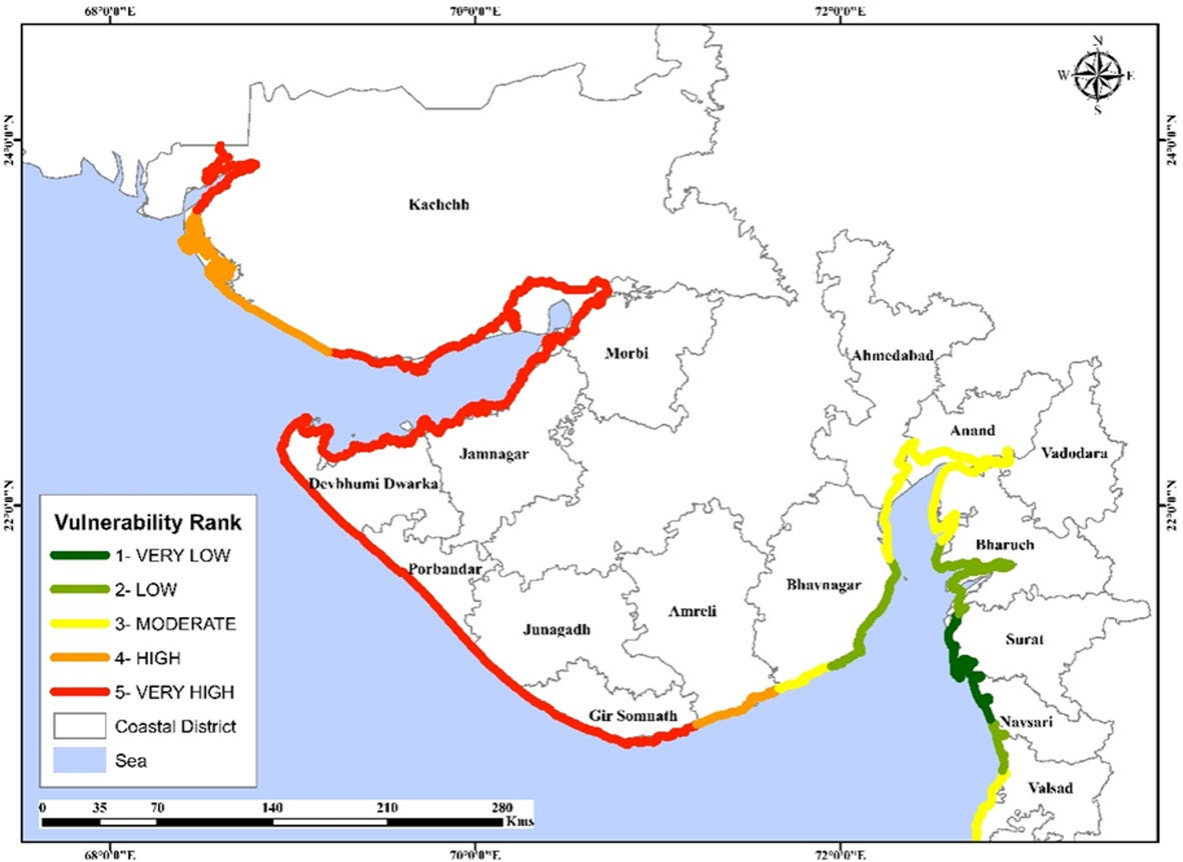
Coastal vulnerability map based on significant wave height for the Gujarat coast. This figure was created by authors using ArcGIS 10.3.1 version on the Google Pro maps.

Since wave energy responsible for sediment transport increases with the square of the significant wave height, the significant wave height helps determine the area more susceptible to erosion^60^. The wave height analysis reveals that 50.21% of transects along the Gujarat coast fall under the very high vulnerability category, indicating widespread exposure to intense wave energy. As shown in Fig. 12, wave action significantly influences shoreline dynamics, with 13.15% of transects classified as highly vulnerable and 15.60% as moderately vulnerable. In contrast, only 10.71% and 10.34% of transects are categorised as low- and very-low-vulnerability, respectively. This suggests that calmer wave conditions are confined to limited coastal stretches, while the majority of the shoreline experiences persistent and potentially erosive wave forces.

### Land use land cover

**Fig. 13:**
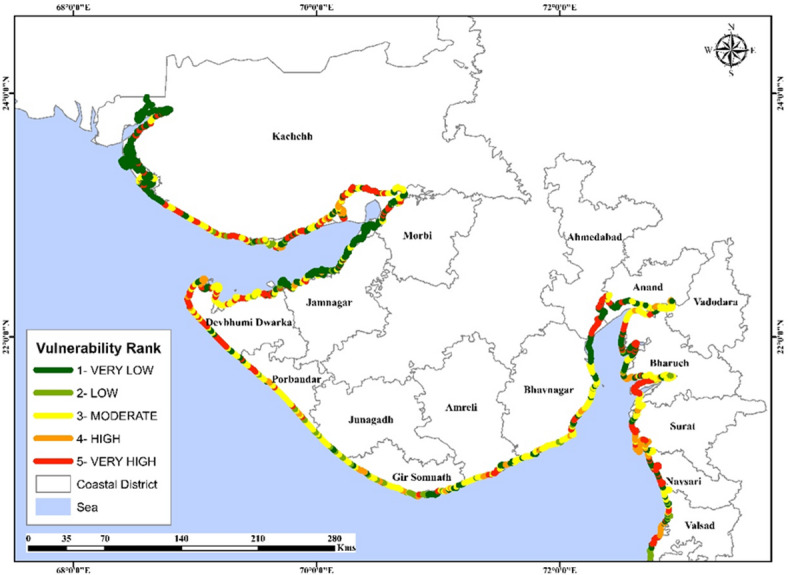
Coastal vulnerability map based on coastal LULC for the Gujarat coast. This figure was created by authors using ArcGIS 10.3.1 version on the Google Pro maps.

GIS techniques can be used to measure these kinds of changes, even if the resulting spatial datasets differ in scale or resolution^78^. The LULC-based coastal vulnerability analysis reveals that 24.57% of transects fall under very high vulnerability, while 26.65% are moderately vulnerable (Fig. 13). Only 29.75% are categorized as very low vulnerability. This distribution emphasises the dominance of moderate to high-risk zones, indicating a significant impact of land-use patterns on coastal stability.

### Population density

**Fig. 14:**
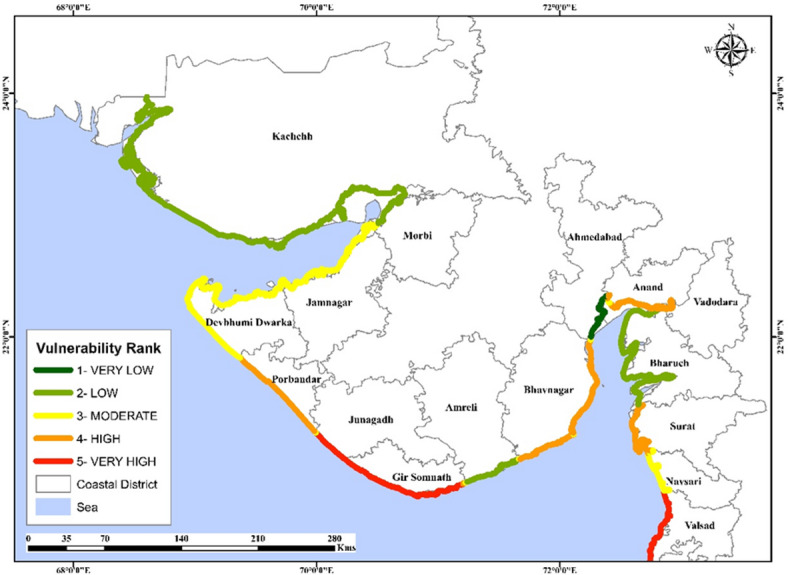
Coastal vulnerability map based on population density for the Gujarat coast. This figure was created by authors using ArcGIS 10.3.1 version on the Google Pro maps.

According to the population density vulnerability analysis, 41.33% of the coastal transects are classified as low vulnerability, indicating areas that are less urbanised or sparsely populated. 21.55% of the segments have a moderate vulnerability rating. However, there is substantial population pressure on 29.5% of the coastline (18.37% high and 11.13% very high), which raises the risk of coastal hazards (Fig. 14). Very low vulnerability is applied to just 7.62% of the territory. These results imply that although a large portion of the coastal stretch is sparsely populated, other regions are at higher risk due to their dense populations and require targeted management approaches.

#### Comparative analysis of various parameters

**Fig. 15:**
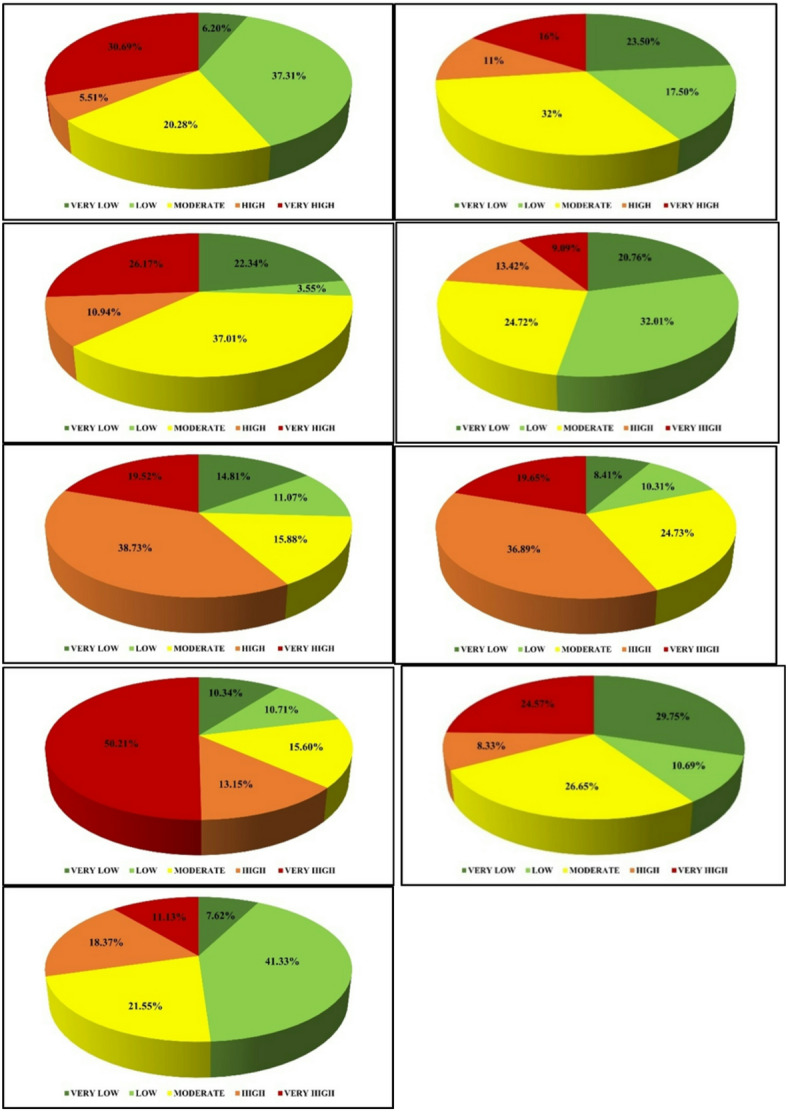
Vulnerability level of (a) Coastal Geomorphology, (b) Coastal Erosion Rate, (c) Sea-level Rise, (d) Mean Tidal Range, (e) coastal elevation, (f) coastal Slope, (g) Significant Wave Height, (h) Coastal LULC, and (i) Population Density Pie charts indicate the percentage occupied by each rank, while the number of transects for each rank are in parentheses.

The comprehensive analysis revealed significant variations in the distribution of risk zones across the different parameters assessed, as illustrated in the pie charts presented in Fig. 15. Among all the contributing factors, the coastal region showed the highest vulnerability to sea level rise, slope, and coastal elevation. These physical parameters significantly influence a region’s susceptibility to long-term inundation, storm surge impacts, and erosion. In contrast, the rate of shoreline change accounted for a comparatively smaller share of the total vulnerability spectrum, suggesting that shoreline movement alone may not fully capture the underlying risks across the region. Other key parameters, such as coastal geomorphology, tidal range, and significant wave height, exhibited a more balanced distribution across all five vulnerability classes. This heterogeneity reflects the diverse physical makeup of the Gujarat coastline, shaped by both natural forces and anthropogenic influences. Furthermore, socio-economic indicators such as land use, land cover, and population density added further layers of complexity to the vulnerability assessment. These factors help highlight areas where human development intersects with environmental fragility, intensifying exposure and potential risk. Overall, the multi-parameter approach underscores the spatial variability of coastal vulnerability in the study area. It confirms the necessity for tailored adaptation strategies that respond to both biophysical conditions and human-induced pressures across different segments of the coastline.

**Fig. 16:**
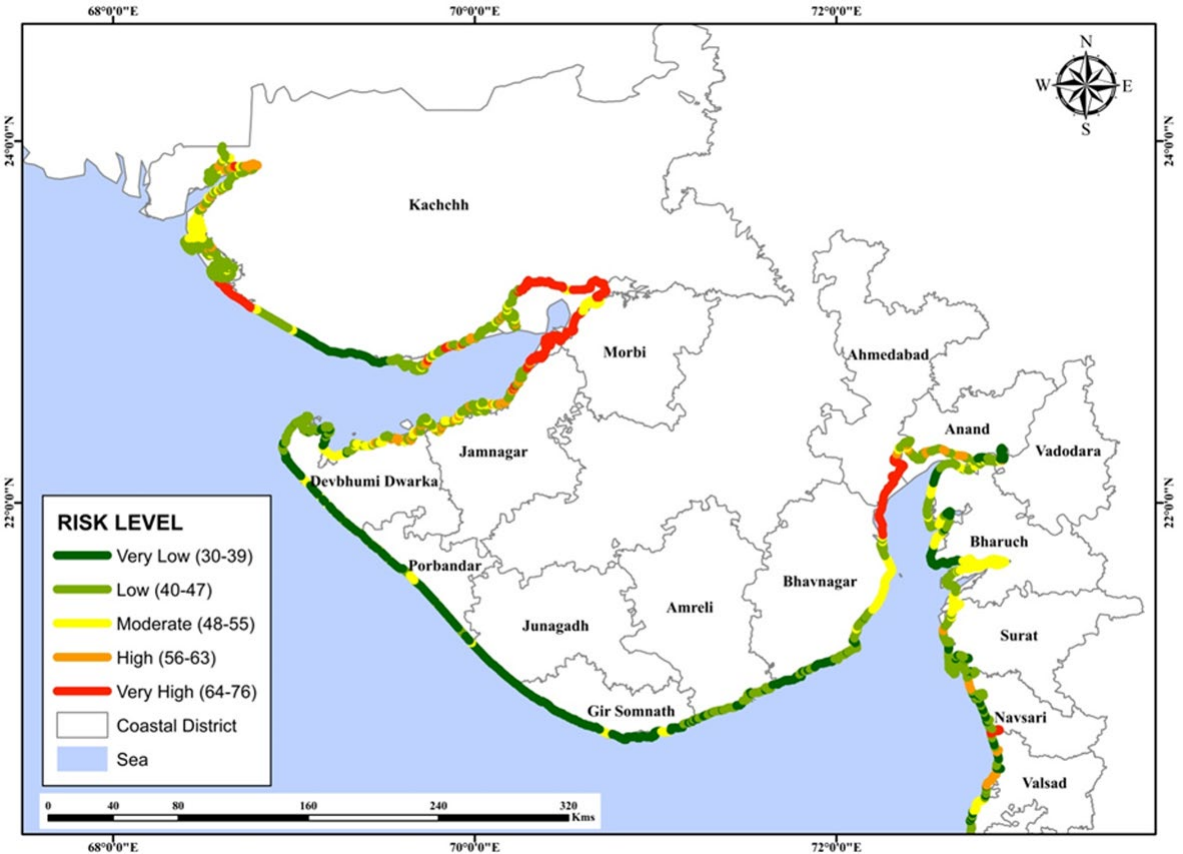
Risk levels map of the Gujarat coast. This figure was created by authors using ArcGIS 10.3.1 version on the Google Pro maps.

#### Coastal vulnerability index

The overall coastal vulnerability was assessed by integrating all contributing input parameters, resulting in a spatial classification of five distinct risk zones. Figure 15 illustrates the proportional distribution of these zones along the Gujarat coastline. Notably, the central coastal stretches, particularly across parts of Anand, Bhavnagar, and Morbi districts, fall within the high and very high vulnerability categories, depicted in red and orange. These areas face heightened exposure due to a combination of factors, including low elevation, steeper coastal slopes, intense wave energy, wide tidal ranges, and significant anthropogenic pressures. The presence of industrial corridors, ports, and expanding urban areas further amplifies susceptibility to hazards such as saline intrusion, shoreline retreat, and the long-term consequences of sea-level rise. In contrast, coastal segments in the northwestern and southern regions, including areas near Kachchh, Devbhumi Dwarka, Porbandar, and southern Gir Somnath, exhibit lower vulnerability (shown in green). These zones benefit from extensive mudflat and beach systems, limited infrastructure development, and relatively stable geomorphological conditions. As a result, they are less prone to immediate coastal threats and demonstrate greater resilience against both natural processes and human-induced stresses, making them potential reference areas for sustainable coastal zone management practices (Fig. 16).

**Fig. 17:**
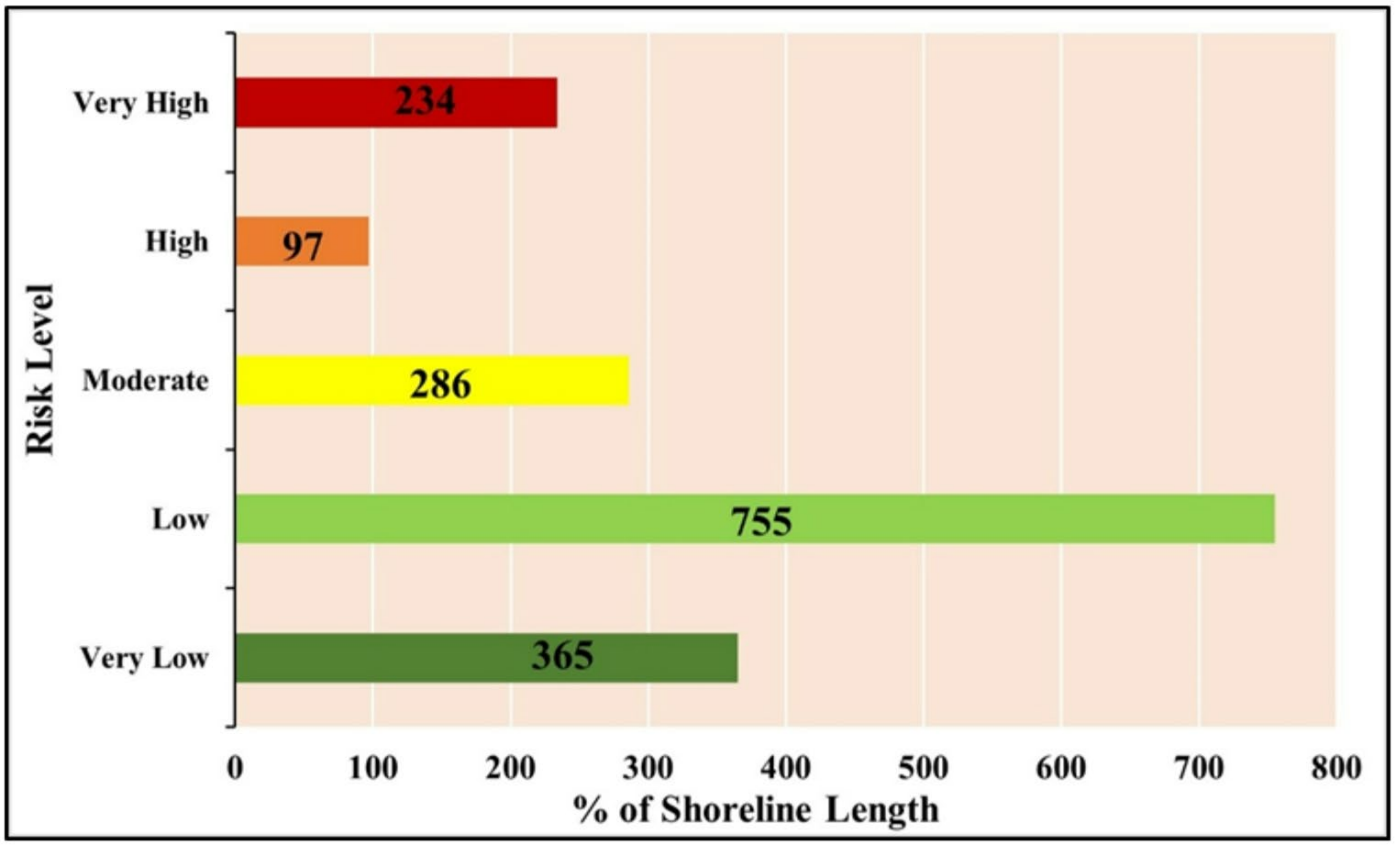
Percentage of shore length for different risk levels of the Gujarat coast.

**Fig. 18:**
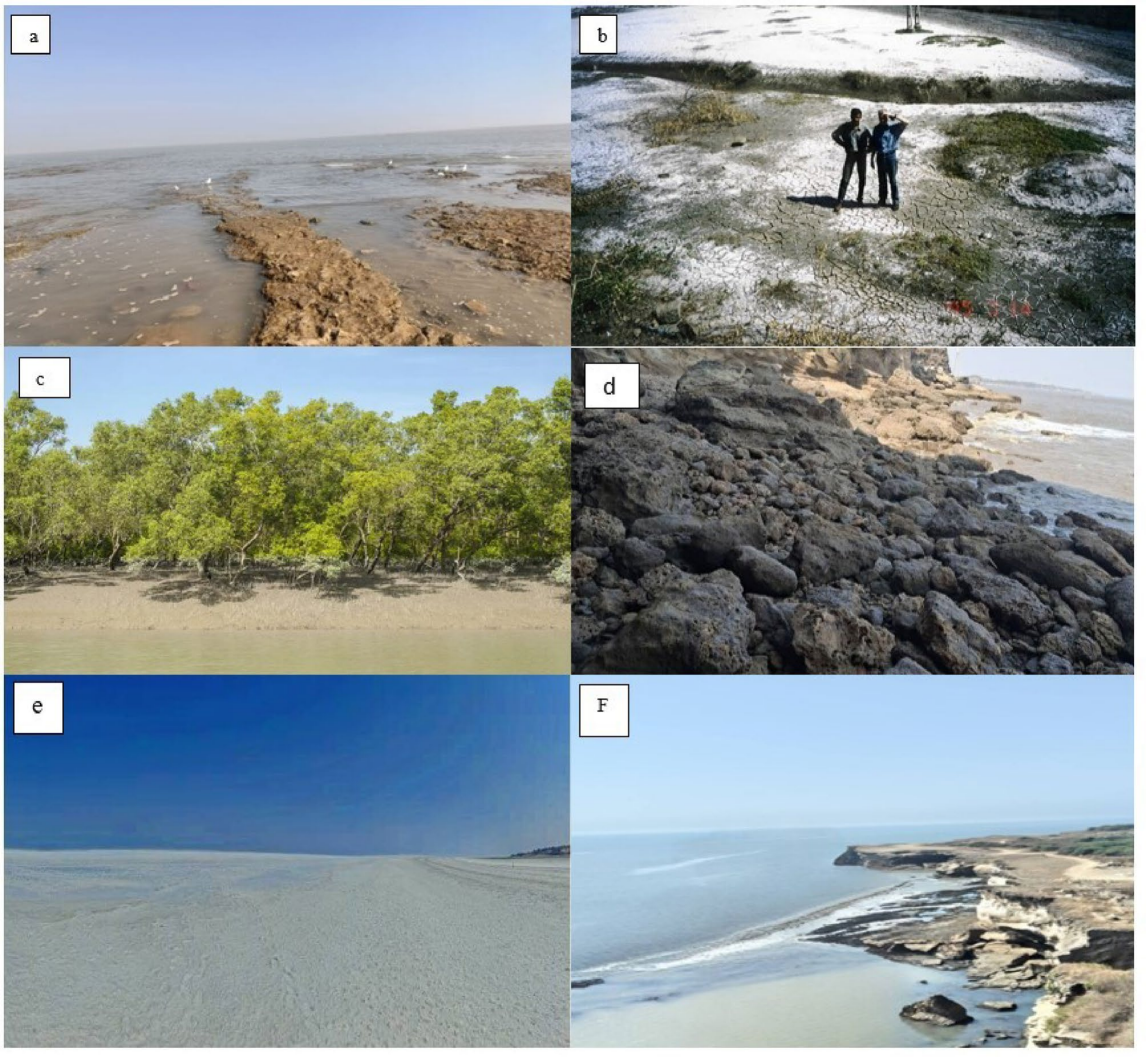
Field Visits: (a). Mud-covered intertidal surface along the Gulf of Khambhat–Saurashtra coast observed during low-tide conditions (b) Salt encrusted mudflat with creek at the background near Dholera, Gulf of Khanbhat (c) Mangrove Forest cover along the Purna River estuary Navsari South Gujrat (d) Erosional Coast cliff along the Bhavnagar coast (e) Salt encrusted surface in Rann of kutch (f) Active coastline erosion due to wave and Tide action along the Diu coastal region.

Figure 17 presents the spatial distribution of Coastal Vulnerability Index (CVI) risk classes based on coastline length. Approximately 755 km of Gujarat’s coastline is classified as low risk, and an additional 365 km falls under the very low-risk category, suggesting that over 60% of the shoreline currently exhibits relative stability. However, this stability is offset by the 234 km of coastline identified as very high risk, highlighting critical zones that demand immediate and long-term resilience planning. Furthermore, 97 km and 286 km of the coastline are classified as high- and moderate-risk areas, respectively. These segments serve as potential early warning areas where vulnerability may increase due to unchecked development, climate change, or coastal system degradation, necessitating proactive monitoring and targeted intervention (Fig. 18).

#### Field observation

## Discussion

The present study offers a detailed spatio-temporal assessment of coastal hazard vulnerability along the Gujarat coastline by integrating long-term shoreline dynamics with key physical and socio-economic parameters within a spatial Coastal Vulnerability Index (CVI) framework. By combining shoreline change rates derived from DSAS with elevation, coastal slope, tidal range, wave height, land-use characteristics, and population distribution, the assessment captures both the intrinsic sensitivity of the coastal system and the magnitude of anthropogenic exposure. The extended temporal coverage from 1990 to 2025, together with a dense and uniformly distributed transect network, enables a more robust representation of spatial heterogeneity in coastal response compared to earlier studies focusing Gujarat coastline that relied on shorter time windows or localized shoreline segments^90,91^. The class-wise distribution of vulnerability shows that very high-vulnerability segments account for approximately 13.47% (∼234 km) of the Gujarat coastline. These sectors are predominantly associated with low-lying topography, gentle coastal gradients, wide tidal ranges, and sustained wave forcing conditions that collectively amplify susceptibility to shoreline instability, tidal inundation, and episodic flooding. Similar associations between low elevation, macrotidal influence, and enhanced coastal risk have been documented along the Saurashtra and central Gujarat coast by Mahapatra et al.^54^ and^60^, particularly in relation to storm surge amplification and tidal inundation potential. The present findings further corroborate these observations by demonstrating that such physical controls remain dominant even when shoreline change is not uniformly erosional. An additional 5.58% (∼97 km) of the coastline falls under the high vulnerability category, where natural sensitivity is compounded by intensive human intervention, including port infrastructure, industrial corridors, and rapidly expanding urban settlements. Studies focusing on shoreline dynamics and land-use transformation along the South Gujarat coast have similarly emphasised the role of coastal modification in intensifying erosion hotspots and destabilising nearshore sediment regimes^90^^,92^. The overlap between high-vulnerability zones and major industrialised coastal belts identified in this study underscores the cumulative impact of physical exposure and socio-economic pressure, a pattern also noted in analytical hierarchy process (AHP)-based vulnerability assessments for Gujarat^54^. Spatially, the pronounced concentration of high and very high vulnerability classes along the central Gujarat coast, particularly within Bhavnagar, Anand, and Morbi districts, reflects the dominant influence of the macrotidal regime of the Gulf of Khambhat. Large tidal amplitudes, extensive low-relief coastal plains, and intense economic activity collectively enhance hazard exposure in this region. Comparable spatial patterns have been consistently reported in earlier assessments^55,60,61^, in which the Gulf of Khambhat emerges as one of the most sensitive coastal sectors of India due to its geomorphic configuration and tidal dominance. The convergence of results across independent studies, despite differences in datasets, spatial resolution, and methodological frameworks, strengthens confidence in the robustness of the vulnerability patterns identified in the present work. Moderate vulnerability zones, accounting for approximately 16.47% of the coastline, represent transitional coastal stretches that currently exhibit partial geomorphic stability but remain susceptible to future perturbations driven by sea-level rise, shoreline modification, and land-use change. Long-term shoreline change studies along Gujarat and adjoining coasts indicate that such transitional zones often experience decadal shifts between accretion and erosion, influenced by sediment redistribution and episodic hydrodynamic forcing^93^. These findings suggest that moderate vulnerability sectors should be considered dynamic rather than stable, particularly under projected climate-driven sea-level rise scenarios. In contrast, the low and very low vulnerability classes together comprise approximately 64.48% of the Gujarat coastline, indicating relative stability across large segments of Kachchh, Devbhumi Dwarka, Porbandar, and southern Gir Somnath. These regions are characterised by comparatively higher elevations, stable geomorphic units such as extensive mudflats and wide beaches, and lower population density. Similar zones of reduced vulnerability and shoreline stability have been reported in DSAS-based and CVI studies along the western coast of India, where limited anthropogenic disturbance and favourable geomorphic settings contribute to lower hazard exposure^56,92,91^.

*Previous studies have documented significant shoreline dynamics along the Gujarat coast using multi-temporal satellite imagery and statistical shoreline analysis approaches. Long-term assessments (1978–2020) indicate that approximately 723.6 km (45.9%) of the Gujarat coastline experienced erosion, with the Kachchh district showing the highest erosion intensity*^92^*. Regional analyses using Digital Shoreline Analysis System (DSAS) Tool with Sentinel-2 and LISS-IV imagery (2012–2021) along the Dahej coast reported maximum erosion rates of 33 m (EPR- End Point Rate), 31 m (LRR- Linear Regression Rate), and 31 m (WLR-Weighted Linear Regression rate), while accretion ranged between 38 and 51 m at specific transects*^94^*. Local-scale studies further reported shoreline retreats of 1590.5 m between 1978 and 2018, with an average rate of 39.76 m yr*^–1^*, indicating accelerated erosion after 1989*^91^*. A broader assessment using Landsat data (1993–2023) showed that 49.7% of transects exhibited accretion, 23.6% erosion, and 26.7% remained stable, highlighting the spatial heterogeneity of coastal changes*^56^.

However, groundwater-focused investigations along the Gujarat coast caution that even morphologically stable sectors may remain vulnerable to indirect hazards such as seawater intrusion under sustained sea-level rise and groundwater extraction^95,96^. The application of the Digital Shoreline Analysis System (DSAS) across 2169 transects spanning 35 years (1990–2025) enables a more detailed characterization of spatial variability in shoreline behaviour than earlier studies conducted over shorter temporal or spatial extents. This shoreline change analysis further reveals that accretion and relative stability dominate substantial portions of the Gujarat coast, particularly in central and northern sectors. Although localized erosion hotspots persist near ports, tidal inlets, and heavily engineered shorelines, erosion is not spatially uniform. This observation aligns with multi-decadal shoreline assessments for Gujarat and other states, such as Odisha, which emphasise strong spatial variability in erosion-accretion trends, governed by geomorphic setting and human intervention rather than regional-scale erosion dominance^93^. The contrast between the present findings and earlier reports of dominant erosion along parts of South Gujarat highlights the importance of long-term datasets and dense transect spacing for capturing evolving shoreline responses beyond short-term variability.

Validation of the CVI results is supported through both external comparison and internal consistency among contributing parameters and field visits. Externally, the close agreement between the spatial distribution of high-risk zones identified in this study and those reported in earlier coastal vulnerability, storm surge, and shoreline change investigations provides independent corroboration of the results^60,61^.

Internally, the systematic alignment between composite vulnerability classes and controlling physical and socio-economic factors further confirms the reliability of the framework adopted to date. High- and very-high-vulnerability zones consistently coincide with low elevation, gentle slopes, strong tidal influence, elevated wave energy, and positive sea-level trends, and also overlap with densely populated and built-up areas. Overall, the findings indicate that sea-level rise, elevation, and coastal slope exert stronger control on vulnerability patterns along the Gujarat coastline than shoreline change rate alone. This reinforces the necessity of integrated assessments that move beyond erosion-centric interpretations and explicitly account for hydrodynamic forcing and human exposure. The identification of highly vulnerable districts such as Bhavnagar, Anand, and Morbi underscores the need for targeted adaptation and risk-reduction strategies. In contrast, relatively stable regions in Kachchh and southern Saurashtra may be better suited to conservation-oriented, sustainable coastal management. The consistency of results with previous Gujarat-coastline studies and the demonstrated internal coherence of the CVI framework confirm the applicability of the proposed methodology for long-term coastal planning and hazard mitigation along the Gujarat coastline.

## Conclusions

This study offers a detailed geospatial assessment of coastal vulnerability along the Gujarat coastline by integrating both physical and socio-economic factors within the Coastal Vulnerability Index (CVI) framework. Utilizing advanced geospatial tools such as the Digital Shoreline Analysis System (DSAS), the research analyzes long-term shoreline change patterns from 1990 to 2025, in conjunction with parameters like elevation, wave height, tidal range, slope, and land use/land cover. The outcome is a spatially nuanced vulnerability profile that captures the complex interplay between environmental dynamics and human influence. The results show that a substantial portion of Gujarat’s coast about 61.55% of the transects is currently experiencing accretion, reflecting areas of natural sediment deposition and relative stability. However, this positive trend is counterbalanced by localized erosion hotspots, particularly along central and southern coastal districts such as Bhavnagar, Anand, and Morbi. These areas exhibit significant shoreline retreat, in some cases exceeding 13 kms, and are subject to both intense human activities and natural geomorphological sensitivity. Industrial expansion, infrastructure development, and population growth amplify these vulnerabilities, positioning these regions as high-priority zones for immediate mitigation and management.

The multi-criteria CVI model used in the study incorporates critical variable, including shoreline change rate, sea level trends, coastal slope, tidal dynamics, wave height, geomorphology, land use, and population density. This comprehensive approach allows for a deeper understanding of the spatial distribution of coastal sensitivity and highlights areas of overlapping physical fragility and socio-economic exposure. While large segments of the coastline fall into moderate or low-vulnerability categories, the persistence of high-risk zones underscores the urgency of sustained monitoring and adaptive management. Importantly, the analysis underscores the spatial heterogeneity of coastal vulnerability across Gujarat. Low-lying, densely populated areas with gentle coastal slopes and high wave activity are especially at risk from sea level rise, storm surges, and environmental degradation. In contrast, regions with higher elevations and more stable landforms exhibit lower sensitivity but still require proactive planning to enhance long-term resilience.

This study serves as a critical decision-support tool for coastal planners, policymakers, and environmental managers. By offering a data-driven, location-specific understanding of vulnerability, it supports targeted interventions and sustainable coastal development. Ultimately, the findings contribute to building climate resilience and improving preparedness for future coastal hazards in Gujarat.

## Data Availability

First author and corresponding author will share on request data that support the study conclusions after a reasonable request.
